# Ultrasensitive ELISA for Accurate Detection of *Plasmodium falciparum* Infection

**DOI:** 10.1093/ofid/ofaf711

**Published:** 2025-11-19

**Authors:** Yuki Kobayashi, Kyo Okita, Po-Kai Chen, Mitsumasa Hasunuma, Taisei Tsuneki, Eri Saki H Hayakawa, Teruki Yoshimura, Etsuro Ito

**Affiliations:** Department of Biology, TWIns, Waseda University, Tokyo, Japan; R&D Department, BioPhenoMA Inc., Waseda University Entrepreneurship Center, Tokyo, Japan; Department of Biology, TWIns, Waseda University, Tokyo, Japan; R&D Department, BioPhenoMA Inc., Waseda University Entrepreneurship Center, Tokyo, Japan; Department of Biology, TWIns, Waseda University, Tokyo, Japan; Department of Biology, TWIns, Waseda University, Tokyo, Japan; Department of Biology, TWIns, Waseda University, Tokyo, Japan; Division of Medical Zoology, Department of Infection and Immunity, Jichi Medical University, Tochigi, Japan; R&D Department, BioPhenoMA Inc., Waseda University Entrepreneurship Center, Tokyo, Japan; School of Pharmaceutical Sciences, Health Sciences University of Hokkaido, Hokkaido, Japan; Department of Biology, TWIns, Waseda University, Tokyo, Japan; R&D Department, BioPhenoMA Inc., Waseda University Entrepreneurship Center, Tokyo, Japan; Graduate Institute of Medicine, Kaohsiung Medical University, Kaohsiung, Taiwan

**Keywords:** malaria, PfHRP2, *Plasmodium falciparum*, pLDH, ultrasensitive ELISA

## Abstract

**Background:**

Rapid and accurate diagnostic methods are crucial for curbing the spread of malaria. Antigen-based rapid diagnostic tests are highly valued for their simplicity, but improved sensitivity is needed for more accurate detection. Furthermore, cases of infection with HRP2-deficient parasites are evading testing, meaning patients are not receiving treatment.

**Methods:**

We developed an ultrasensitive protein detection system for *Plasmodium falciparum* (Pf) histidine-rich protein 2 (PfHRP2) and pan-lactate dehydrogenase (pLDH) by combining an ELISA and a thio-NAD cycling (TN-cyclon™). The reason for attempting to detect pLDH is to accommodate HRP2-deficient strains. The samples measured included recombinant protein in BSA buffer, Pf-parasitized erythrocytes in BSA buffer, and Pf-parasitized erythrocytes in non-infected human whole blood.

**Results:**

Our technology detected recombinant PfHRP2 and pLDH at concentrations as low as 0.782 pg/mL and 1.33 pg/mL, respectively. This system detected *Plasmodium falciparum* parasitemia in *in vitro* culture at levels as low as 2.57 × 10^−5^% for PfHRP2 and 2.41 × 10^−5^% for pLDH. We then mixed purified parasitized erythrocytes with human whole blood to mimic whole blood conditions. Parasitemia of Pf-parasitized erythrocytes in human whole blood was successfully detected at levels as low as 0.5 parasites/μL for both PfHRP2 and pLDH.

**Conclusions:**

The detection sensitivity of our system is approximately 10 times greater than that of the latest ultrasensitive antigen detection kits. Our results demonstrate that our developed assay requires no additional processing to separate parasitized erythrocytes from whole blood samples and that the *pfhrp2*-gene deletion strain can be successfully detected using the pLDH-based TN-cyclon™ test.

## Teaser

Our technology, TN-cyclon™, can detect PfHRP2 and pLDH with a detection sensitivity that is 10 times higher than that of the latest ultrasensitive antigen detection kits. It is useful for HRP2-deficient strains due to its ability to detect pLDH.

## Graphical Abstract

**Graphical Abstract:**
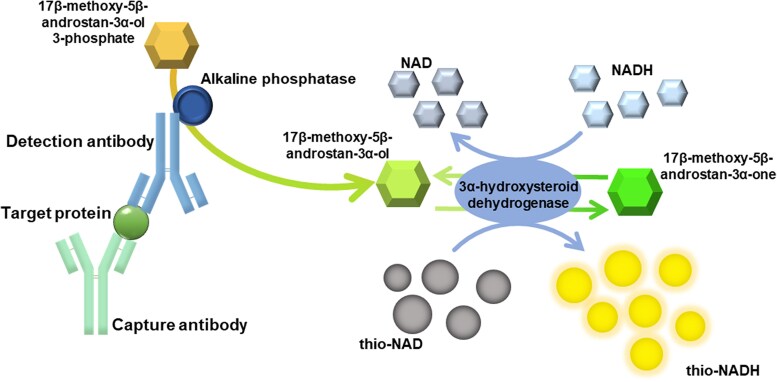

Malaria is caused by *Plasmodium* spp. infection transmitted through the bite of female *Anopheles* mosquitoes and is prevalent in tropical and subtropical areas of the world. *The World Malaria Report 2024* from the World Health Organization (WHO) estimated 263 million malaria cases in 2023, representing an increase of 11 million cases compared with 2022 [1]. Five species of *Plasmodium* parasites are known to cause malaria in humans: *Plasmodium falciparum* (*P falciparum*), *Plasmodium vivax*, *Plasmodium ovale*, *Plasmodium malariae*, and *Plasmodium knowlesi*. *P knowlesi* is a zoonotic parasite that also causes malaria in monkeys [2]. Among the 5 *Plasmodium* species, *P falciparum* is the most lethal, causing high fever, cerebral infarction, and coma [3], and can lead to death without early diagnosis and appropriate treatment.

Microscopic examination of thick and thin blood smears is the gold standard method for diagnosing malaria, as it is the most cost-effective and straightforward diagnostic method available [4]. Both the need for highly trained and skilled microscopists and insufficient sensitivity for detecting low levels of parasitemia, however, limit its usefulness [5]. In addition, because *P falciparum* erythrocytes sequester in the organ microvasculature during the trophozoite and schizont stages of the asexual life cycle, they evade detection by conventional blood smear analysis [6]. Therefore, the ability to directly measure substances derived from the parasite is a compelling strategy. PCR-based methods are highly sensitive and capable of distinguishing between the 5 *Plasmodium* species. However, PCR has several issues [7]. Among PCR-based methods, species-specific differentiation techniques targeting rRNA genes have been developed [8]. Furthermore, to minimize the likelihood of false-negative results associated with the commonly targeted antigen *P falciparum* histidine-rich protein 2 (PfHRP2), a novel detection system has been developed by incorporating the *P falciparum* homolog of insulin-degrading enzyme (PfIDEh) as a potential alternative biomarker in PCR-based assays [9]. Although PCR techniques have high sensitivity, they require sophisticated equipment, air-conditioned rooms and trained personnel, which presents significant barriers to their adoption in malaria-endemic regions.

PCR has certain limitations and practical challenges to begin with [7]. As noted by Wang et al [10], PCR assays are susceptible to both nonspecific (ie, false-positive) and false-negative amplifications. Moreover, the small sample volume that can be processed, typically at the microliter scale, makes it difficult to consistently obtain target nucleic acids, thereby compromising assay reliability. The PCR technique is also time-consuming, costly, and technically demanding, and the enzymes involved in amplification may become inactivated during the reaction. A critical concern is that nucleic acids may remain detectable even after parasites have been eliminated through therapeutic intervention, which can lead to false-positive results, a phenomenon that has also been observed in *Mycobacterium tuberculosis* infections [10]. These limitations underscore the need to develop ultrasensitive diagnostic methods that target proteins rather than nucleic acids, since protein-based detection is more likely to reflect the presence of viable parasites.

Rapid diagnostic tests (RDTs), which are lower in cost, faster, and more convenient than PCR, have become widely utilized for detecting malaria-specific biomarkers (ie, proteins) such as PfHRP2 and pan lactate dehydrogenase (pLDH), with approximately 3.9 billion units sold globally between 2010 and 2022 [11]. The biomarker PfHRP2 is a water-soluble protein uniquely expressed by *P falciparum* [12]. During the intraerythrocytic stages, PfHRP2 localizes to the erythrocyte membrane and is expressed from the asexual life cycle to the immature gametocyte stage [12–14]. On the other hand, pLDH is a metabolic enzyme in malaria parasites that functions as a glycolytic enzyme relying on ATP production through glucose metabolism [15]. Unlike PfHRP2, pLDH is expressed by all 5 species of human-specific malaria parasites, with Pf-pLDH and pLDH from other species sharing 90%–92% sequence identity [15]. Whereas PfHRP2 excels at identifying *P falciparum*-specific infections, pLDH is rapidly cleared from the bloodstream in parallel with parasite clearance, making it particularly suitable for diagnosing real-time infection status [16, 17]. These tests, based on immunochromatography, are highly valued for their simplicity and efficiency, making them particularly suitable for field applications. The diagnostic accuracy of RDTs, however, varies considerably across different manufacturers [18]. Notably, in the United States, only a single RDT has received regulatory approval, reflecting the stringent requirements for diagnostic reliability [2]. In terms of detection sensitivity, early antigen tests could detect only several tens of parasites per microliter of blood [19]. Even the most recent assays, which are considered highly sensitive, achieve detection limits of only a few parasites per microliter [20].

Aside from the importance of accuracy and detection sensitivity of the RDT development, asymptomatic carriers of malaria are also a big problem for accurately detecting malaria-positive carriers. Some asymptomatic malaria carriers can evade detection by RDTs and hematologic identification by microscopy, even when PCR is positive, leading to a high rate of false negatives [21]. It is important to note that PCR can also detect dead parasites, so a positive PCR result does not necessarily indicate a correct diagnosis. In areas with high rates of malaria infections, asymptomatic malaria carriers often remain untreated, which hinders efforts to eradicate this disease. Identifying and treating positive malaria parasite carriers is crucial for eliminating malaria. In light of the present situation, improving the accuracy and sensitivity of antigen-based diagnostic tests such as RDTs and promoting their widespread implementation is critical for the timely identification, effective treatment, and overall control of malaria.

In the present study, to increase the detection sensitivity of antigen tests and develop ultrasensitive RDTs in the future, we employed the TN-cyclon™, an approach we developed for protein detection, enabling ultrahigh sensitivity [22], rapidity, and simplicity. The TN-cyclon™ combines the principles of a sandwich ELISA with the thio-NAD cycling method, enabling signal amplification for protein quantification [23, 24]. In this system, a sandwich ELISA is performed using a detection antibody labeled with alkaline phosphatase (ALP), and signal amplification is achieved through thio-NAD cycling, involving 3α-hydroxysteroid dehydrogenase (3α-HSD) as the enzyme and thio-NAD and NADH as the coenzymes. Although proteins cannot be amplified like nucleic acids, our hierarchical sequential thio-NAD amplification system enables the amplification of thio-NADH, which correlates with protein levels, providing a robust tool for protein-based diagnostics with ultrahigh sensitivity. In other words, the TN-cyclon™ method enables ultrasensitive measurements equivalent to those of digital ELISA [25], and if this detection system is replaced with a simpler ELISA system in the future, it is likely to lead to the most highly sensitive RDT.

## METHODS

### Reagents and Chemicals

For PfHRP2 measurement, MAL3-902 and MAL2-1564 were used as the capture and detection antibodies, respectively, and for pLDH measurement, PFL8-883 and PFL9-488 were used as the capture and detection antibodies, respectively. All 4 antibodies were obtained from Bio Matrix Research (Chiba, Japan) and kindly gifted from the AACJ Malaria Consortium Inc. (Tokyo, Japan). The ALP labeling kit used for labeling the detection antibodies was purchased from Dojindo Laboratories (Kumamoto, Japan). PfHRP2 recombinant protein (R01596) and pLDH recombinant protein (BN1126) were purchased from Meridian Bioscience (Cincinnati, OH, United States). NADH for the thio-NAD cycling reaction was purchased from MilliporeSigma (N1161-10VL; St. Louis, MO, United States), thio-NAD from Oriental East (44104001; Tokyo, Japan), and 3α-HSD from Asahi Kasei Pharma (T-58; Tokyo, Japan); a substrate, 17β-methoxy-5β-androstan-3α-ol 3-phosphate, was synthesized by one of the authors (T.Y.) [26].

### Cultivation of *P falciparum*-Parasitized Erythrocytes

Human erythrocytes (O^+^ blood) were obtained from the Japanese Red Cross Society (authorization number: 25J0045). The protocol for *P falciparum* cultivation with human erythrocytes was approved by the Jichi Medical University Bioethics Committee for Medical Research (Certification of Approval number: RIN DAI 18-HEN 011). *P falciparum* (line 3D7) was cultivated as previously described [27, 28]. Briefly, human non-parasitized blood cells (nRBCs) were washed 3 times with incomplete RPMI 1640 (iRPMI), based on RPMI 1640 medium (Invitrogen Life Technologies, Grand Island, NY, United States) [28]. Hematocrit (Ht) was adjusted to 50% with iRPMI and stored at 4°C until samples were used for the experiments. *P falciparum* cultivation was cultured at 3%-Ht using complete RPMI 1640 medium (cRPMI), which consisted of iRPMI medium supplemented with 5 mg/mL AlbuMAX® II Lipid-Rich BSA (Invitrogen Life Technologies), adjusted to pH 7.25–7.4, and sterilized with a 0.22-μm bottle-top filter (Corning, Glendale, AZ, United States).

### Ultrasensitive ELISA Assay by TN-cyclon™

The TN-cyclon™ assay was developed by Ito and colleagues [26]. TN-cyclon™ was designed and developed based on the concept that if it is not possible to amplify and measure proteins themselves, then amplifying the signal used to detect proteins would be feasible [29]. It is based on sandwich ELISA and combines it with thio-NAD cycling. Both sandwich ELISA and thio-NAD cycling only increase the signal linearly. However, combining these 2 techniques enables signal amplification in a triangle number (ie, quadratic fashion), resulting in ultrasensitive measurements in a short time [30]. Previous research results have enabled ultrasensitive diagnostics targeting the MPT64 protein of *M tuberculosis* and ultrasensitive detection of the p24 protein of HIV-1 [10, 31]. In experiments, the wells of an Immuno Module F8 Maxi Soap plate (469949; Thermo Fisher Scientific, Waltham, MA, United States) were coated with 100 µL of capture antibody solution diluted to 10 µg/mL in 50 mM Na_2_CO_3_ buffer (pH 9.6) and incubated for 1 hour at room temperature. Subsequently, 100 µL of 1% BSA in Tris-buffered saline (TBS) was added to each well for blocking, and the plate was incubated at room temperature for 1 hour. After blocking, 100 µL of antigen solution was added, and the plate was shaken at room temperature for 1 hour. The antigen solution, containing both PfHRP2 and pLDH recombinant proteins, was diluted 2-fold in 0.1% BSA in TBS to final concentrations ranging from 1000 pg/mL to 31.25 pg/mL. A detection antibody conjugated with ALP was diluted to 100 ng/mL in TBS containing 0.1% BSA and 0.05% Tween 20, added to wells in 100 µL portions, and incubated with shaking for 1 hour at room temperature. To amplify the ELISA signals, 100 µL of the reaction mixture (100 mM Tris-HCl with 10 U/mL 3α-HSD, 0.4 mM 17β-methoxy-5β-androstan-3α-ol 3-phosphate, 1.0 mM NADH, and 2.0 mM thio-NAD) was added to each well. In the thio-NAD cycling reaction, thio-NADH accumulation was monitored based on the increase in absorbance at 400 nm (11 900 M^−1^ cm^−1^), typically detected at 405 nm using a microplate reader (SH-1000; Corona Electric, Ibaraki, Japan). Signals at 405 nm were normalized to those at 660 nm, and the difference (A_405_–A_660_) was used for statistical analysis. The TN-cyclon™ system was used exclusively for laboratory-based experiments in the present study. The device requires a microplate reader and a refrigerator for reagent storage. A compact prototype microplate reader specifically designed for TN-cyclon™ applications is currently under development, but it was not utilized in the present experiments.

### Limit of Detection of PfHRP2/pLDH in pRBCs With Culture Medium by TN-cyclon™

We evaluated the limit of detection (LOD) for the ratio of parasitized red blood cells (pRBCs) using PfHRP2 or pLDH as indices. To maintain a consistent Ht level throughout the experiment, we prepared 2-fold serial dilutions of cultured pRBCs mixed with nRBCs in iRPMI, with the range of parasitemia dilution varying from 0.1% to 0.000625%. We calculated the number of pRBCs based on the assumption of 5 million erythrocytes per microliter [26]. Given that pRBCs were cultured at an Ht of 3% and a parasitemia of 2.73% before dilution, we determined that there are 4.1 × 10^3^ erythrocytes per microliter in a sample with an infection rate of 2.73%. Using this calculation, we converted the diluted cultured samples of parasitemia ranging from 0.01% to 0.000625% for the TN-cyclon™ assay, resulting in parasitemia from 15 to 0.94 parasites/µL. We used whole stages of pRBCs in culture for the ELISA assay without separation by each infection stage because PfHRP2, a marker Pf protein, is expressed in all asexual stages.

### LOD of PfHRP2/pLDH in pRBCs With Human Non-parasitized Whole Blood by TN-cyclon™

Human non-parasitized whole blood (hereafter referred to as “whole blood”), treated with EDTA-2 K as an anticoagulant, was purchased from BioIVT (CTSAG029020; Hicksville, NY, United States) and confirmed to be negative for hepatitis B virus, hepatitis C virus, HIV-I/II, and syphilis. The pRBCs were mixed with the whole blood at concentrations of 4, 2, 1, and 0.5 parasites/µL, diluted with whole blood. These pseudo blood samples were diluted 100 times with 0.1% BSA in TBS and measured using TN-cyclon™ as described above with antibodies for PfHRP2 and pLDH, respectively. A 100-fold dilution was used here because studies using blood (serum and plasma) demonstrated that spike-recovery test results are nearly 100% [32].

### Statistical Analysis

Data are presented as the mean ± standard deviation. Experimental values were obtained by subtracting the mean absorbance of the blank signals from the corresponding time point and concentration measurements. The LOD was statistically calculated based on the mean and standard deviation of the blank signals, applying a confidence factor of 3. The limit of quantitation (LOQ) was similarly estimated, using a confidence factor of 10. Statistical analysis of differences between groups was performed using a 1-way ANOVA followed by a *post hoc* Holm test in R (version 4.2.2), with *P* < .05 considered statistically significant.

## RESULTS

### LODs for Recombinant PfHRP2 and pLDH Proteins

**Figure 1.**
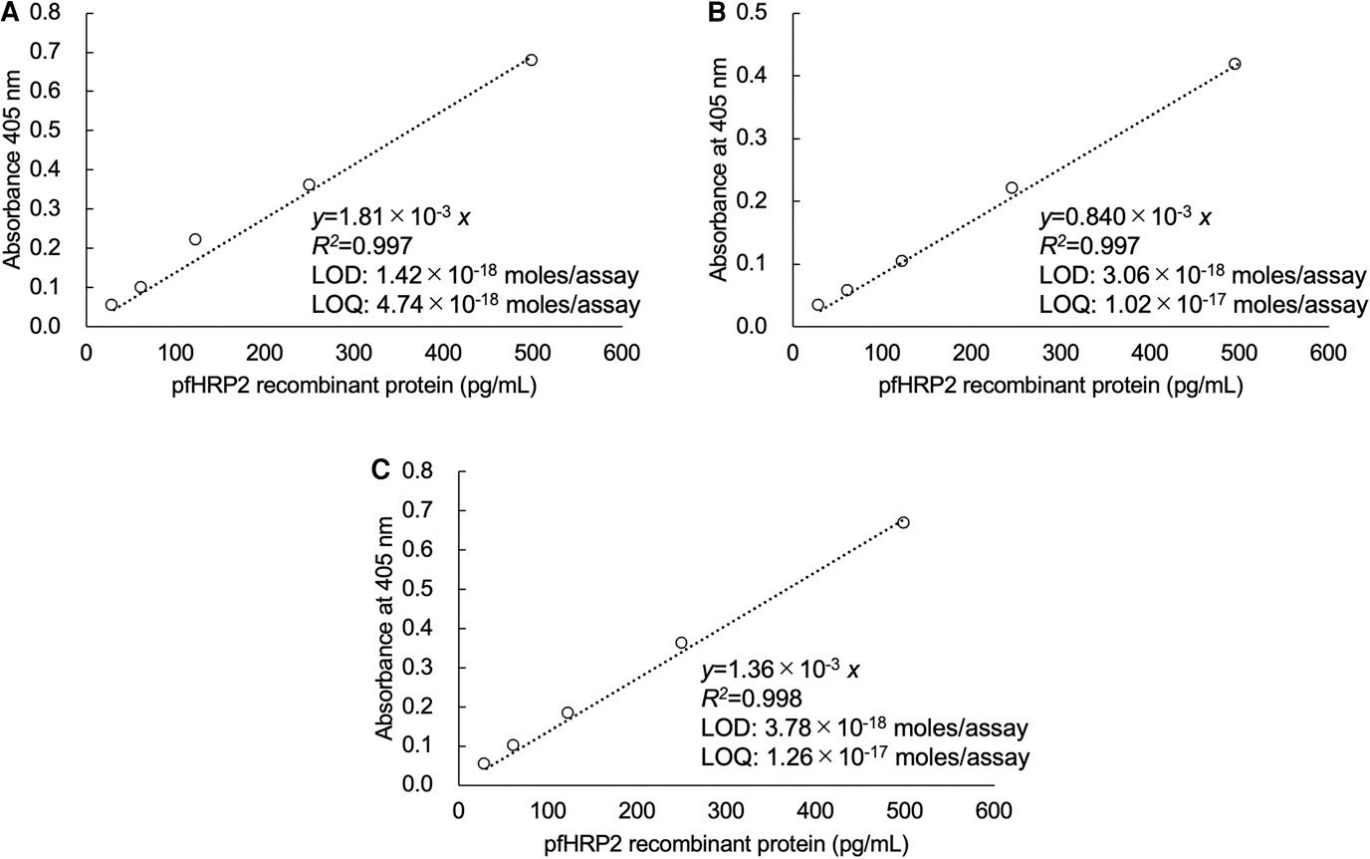
Linear calibration curves of TN-cyclon™ for PfHRP2 recombinant protein. The experiments were conducted by 3 investigators as indicated by (*A*)–(*C*). Absorbance was measured at the following time points of thio-NAD cycling: 35 min for investigator *A*, 60 min for investigator *B*, and 20 min for investigator *C*. The measurement time was selected from each record to provide the most sensitive measurement.

To investigate the detection limit for Pf-protein concentrations using our assay system, we utilized recombinant PfHRP2 or pLDH at various concentrations to assess the detection limits (ie, intra-assay). Furthermore, we examined whether measurement errors occur when tests are conducted by different investigators (ie, inter-assay). For PfHRP2, the linear calibration curves by 3 different investigators were as follows: *y* = 1.81 × 10^−3^*x* (*R*^2^ = 0.997), *y* = 0.840 × 10^−3^*x* (*R*^2^ = 0.997), and *y* = 1.36 × 10^−3^*x* (*R*^2^ = 0.998), respectively (Figure 1*A*–*C*). The LODs were calculated to be 1.42 × 10^−18^ moles/assay, 3.06 × 10^−18^ moles/assay, and 3.78 × 10^−18^ moles/assay, respectively. Considering that the assay volume was 100 µL/well and the molecular weight of PfHRP2 is 55 k, the calculated LODs were 0.782 pg/mL, 1.69 pg/mL, and 2.08 pg/mL by each investigator, respectively (Figure 1*A*–*C*). The LOQs were 4.74 × 10^−18^ moles/assay (2.61 pg/mL), 1.02 × 10^−17^ moles/assay (5.62 pg/mL), and 1.26 × 10^−17^ moles/assay (6.93 pg/mL) by each investigator, respectively (Figure 1*A*–*C*).

**Figure 2.**
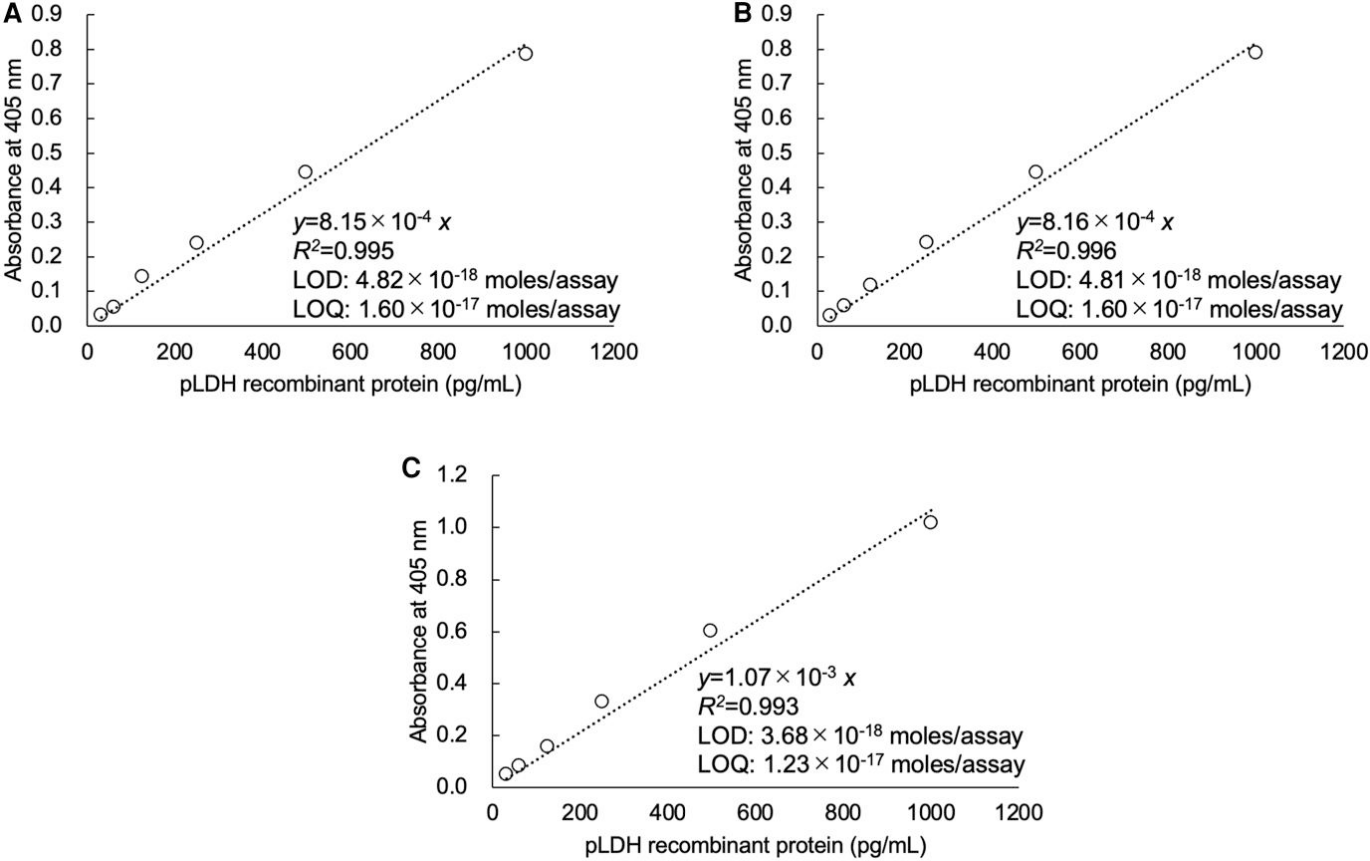
Linear calibration curves of TN-cyclon™ for pLDH recombinant protein. The experiments were conducted by 3 investigators as indicated by (*A*)–(*C*). Absorbance was measured at the following time points of thio-NAD cycling: 30 min for investigator *A* and 60 min for investigators *B* and *C*. The measurement time was selected from each record to provide the most sensitive measurement.

For pLDH, the linear calibration curves obtained by the 3 different investigators were as follows: *y* = 8.15 × 10^−4^*x* (*R*^2^ = 0.995), *y* = 8.16 × 10^−4^*x* (*R*^2^ = 0.996), and *y* = 1.07 × 10^−3^*x* (*R*^2^ = 0.993), respectively (Figure 2*A*–*C*). The LODs were 4.82 × 10^−18^ moles/assay (1.74 pg/mL), 4.81 × 10^−18^ moles/assay (1.73 pg/m), and 3.68 × 10^−18^ moles/assay (1.33 pg/mL), and the LOQs were 1.60 × 10^−17^ moles/assay (5.78 pg/mL), 1.60 × 10^−17^ moles/assay (5.77 pg/mL), and 1.23 × 10^−17^ moles/assay (4.42 pg/mL), respectively (Figure 2*A*–*C*). The molecular weight of pLDH is 36 k. The thio-NAD cycling time was selected to determine the optimal sensitivity (Figure 2*A*–*C*). The LODs for the 2 recombinant proteins reached 10^−18^ moles/assay, demonstrating that they are sufficiently measured with ultrahigh sensitivity [33].

### LODs of pRBCs in Culture Medium Using PfHRP2 and pLDH as Indices by TN-cyclon™

**Figure 3.**
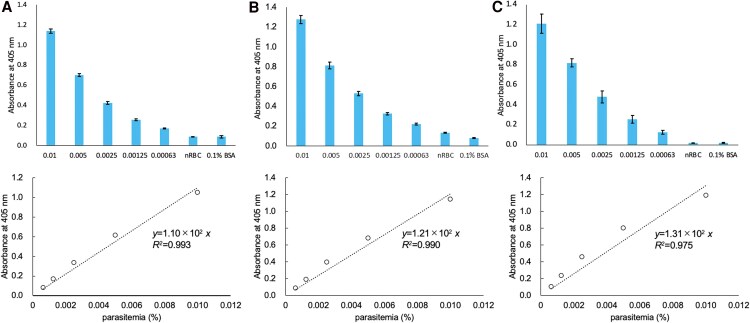
Limits of detection of pRBCs in culture medium using PfHRP2 as the index. Cultured Pf samples were diluted by iRPMI with a consistency of 3% Ht, and parasitemia was set to range from 0.01% to 0.000625%. Three datasets were measured by 3 investigators and are presented as (*A*)–(*C*). nRBC stands for non-parasitized erythrocytes. The control vehicle was 0.1% BSA. Triplicate measurements were performed at 60 min of thio-NAD cycling by each investigator. Data are presented as the mean ± standard deviation. Linear calibration curves were generated by subtracting the absorbance of wells with non-parasitized erythrocytes from the absorbance values recorded by each experimenter (shown in the bottom graph). The LODs and LOQs were 2.57 × 10^−5^% and 8.55 × 10^−5^%, respectively, for (*A*); 6.50 × 10^−5^% and 2.17 × 10^−4^%, respectively, for (*B*); and 1.87 × 10^−5^% and 6.24 × 10^−5^%, respectively, for (*C*).

We evaluated the LOD of the pRBCs in iRPMI using PfHRP2 and pLDH as indices. Cultivated Pf samples, ie, pRBCs, nRBCs, and cRPMI mixture, were diluted by iRPMI to 3% Ht, and parasitemia was set to range from 0.01% to 0.000625%. When using PfHRP2 as an index, the linear calibration curves for parasitemia versus the absorption of thio-NADH obtained by the 3 different investigators were as follows: *y* = 1.10 × 10^2^*x* (*R*^2^ = 0.993) (Figure 3*A*), *y* = 1.21 × 10^2^*x* (*R*^2^ = 0.990) (Figure 3*B*), and *y* = 1.31 × 10^2^*x* (*R*^2^ = 0.975) (Figure 3*C*). Respectively, the LODs were 2.57 × 10^−5^% (4.44 × 10^−2^ parasites/µL) (Figure 3*A*), 6.50 × 10^−5^% (0.112 parasites/μL) (Figure 3*B*), and 1.87 × 10^−5^% (3.23 × 10^−2^ parasites/µL) (Figure 3*C*); the LOQs were 8.55 × 10^−5^% (0.148 parasites/µL) (Figure 3*A*), 2.17 × 10^−4^% (0.374 parasites/μL) (Figure 3*B*), and 6.24 × 10^−5^% (0.108 parasites/µL) (Figure 3*C*).

**Figure 4.**
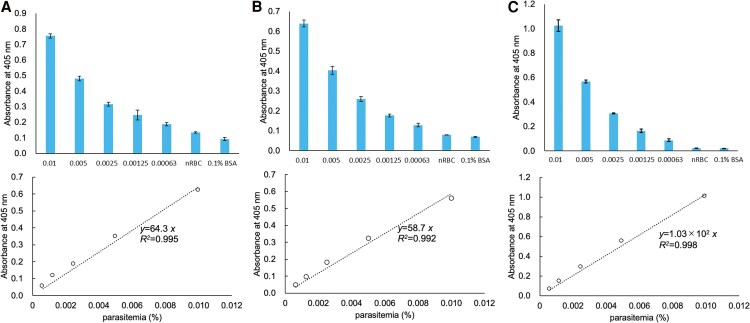
Limits of detection of pRBCs in culture medium using pLDH as the index. Cultured Pf samples were diluted by iRPMI with 3% Ht, and parasitemia was set to range from 0.01% to 0.000625%. Three datasets were measured by 3 investigators and are presented as (*A*)–(*C*). nRBC stands for non-parasitized erythrocytes. The control vehicle was 0.1% BSA. Triplicate measurements were performed at 60 min of thio-NAD cycling by each investigator. Data are presented as the mean ± standard deviation. Linear calibration curves were generated by subtracting the absorbance of wells with non-parasitized erythrocytes from the absorbance values recorded by each experimenter (shown in the bottom graph). The LODs and LOQs were 1.92 × 10^−4^% and 5.80 × 10^−4^%, respectively, for (*A*); 2.41 × 10^−5^% and 8.03 × 10^−5^%, respectively for (*B*); and 2.18 × 10^−5^% and 7.28 × 10^−5^%, respectively for (*C*).

When using pLDH as an index, the linear calibration curves for parasitemia versus the absorption of thio-NADH obtained by the 3 investigators were as follows: *y* = 64.3*x* (*R*^2^ = 0.995) (Figure 4*A*), *y* = 58.7*x* (*R*^2^ = 0.992) (Figure 4*B*), and *y* = 1.03 × 10^2^*x* (*R*^2^ = 0.998) (Figure 4*C*), respectively. The LODs were 1.92 × 10^−4^% (0.331 parasites/µL) (Figure 4*A*), 2.41 × 10^−5^% (3.62 × 10^−2^ parasites/µL) (Figure 4*B*), and 2.18 × 10^−5^% (3.77 × 10^−2^ parasites/µL) (Figure 4*C*), and the LOQs were 5.80 × 10^−4^% (1.10 parasites/µL) (Figure 4*A*), 8.03 × 10^−5^% (0.121 parasites/µL) (Figure 4*B*), and 7.28 × 10^−5^% (0.126 parasites/µL) (Figure 4*C*). We found that our TN-cyclon™ detected pRBCs with ultrahigh sensitivity using either PfHRP2 or pLDH as the index.

### LOD of pRBCs in Non-parasitized Human Whole Blood Using PfHRP2 and pLDH as Indices by TN-cyclon™

**Figure 5.**
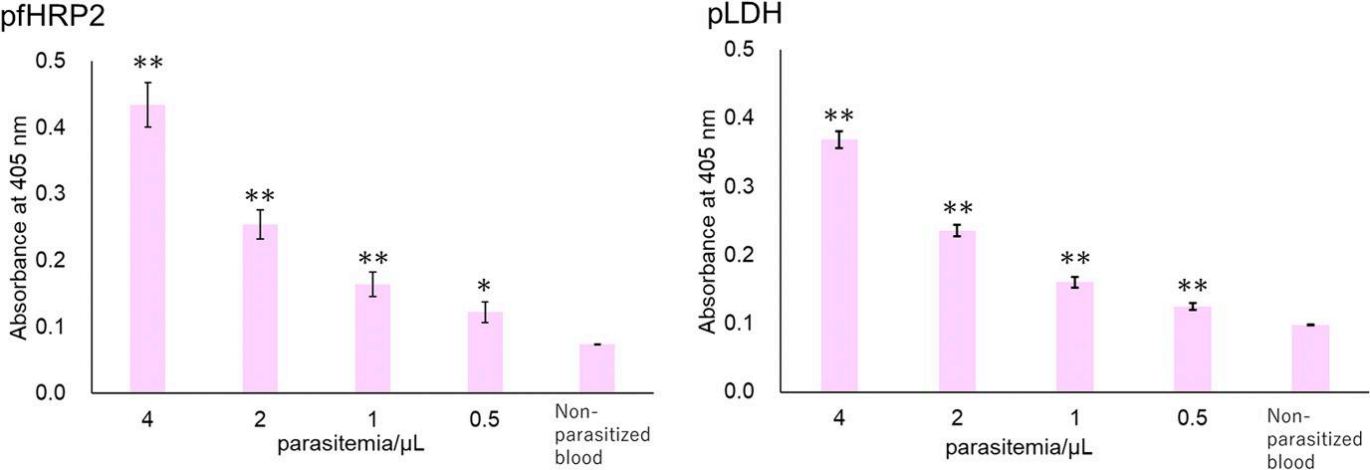
Limits of detection of pRBCs in human whole blood using PfHRP2 or pLDH as the indices. We mixed pRBCs with non-parasitized human blood to create pseudo-malaria-infected blood and detected pRBCs. Triplicate measurements were performed at 60 min of thio-NAD cycling for both antigens. Data are presented as the mean ± standard deviation. Specimens of all concentrations showed that the signals were significantly higher than in the non-parasitized whole blood (*P < .05, **P < .01).

Because human blood contains various proteins, ELISA using blood samples may be affected in some way. Therefore, to confirm whether our TN-cyclon™ system can accurately detect pRBCs in human blood, we mixed pRBCs with non-parasitized human blood to create pseudo-malaria-infected blood and conducted measurements using our TN-cyclon™ method. Using the both PfHRP2 and pLDH indices, the absorbance of pseudo blood samples with concentrations ranging from 4 to 0.5 parasites/µL was significantly different from that of the non-parasitized blood (EDTA-2K-treated human whole blood) (Figure 5). Our method accurately detected parasitemia down to 0.5 parasites/µL, even in whole blood.

## DISCUSSION

In response to the recent emergence of PfHRP2-deficient strains, the WHO recommends testing all consenting suspected malaria cases simultaneously using both WHO-recommended PfHRP2 RDTs and non-HRP2-based methods (eg, pLDH RDTs) [34 ]. To meet this challenge, we successfully developed an ultrasensitive protein detection system for both PfHRP2 and pLDH. The results of the present study demonstrated an LOD of 0.782 pg/mL for PfHRP2 recombinant protein and an LOD of 1.33 pg/mL for pLDH recombinant protein. Given that the typical LODs for commercial RDTs are 800–1000 pg/mL for HRP2 and 25 ng/mL for pLDH [35], the newly developed TN-cyclon™ system showed greater detection sensitivity for PfHRP2 and pLDH compared to the commercially available RDTs. In the laboratory, on the other hand, the method utilizing bead suspension array technology reported by Martiáñez-Vendrell et al [36] showed an LOD of 6.0 pg/mL for PfHRP2 and 56.1 pg/mL for pLDH. An interdigitated electrode sensor for impedimetric detection of PfHRP2 by Soraya et al [37] achieved an LOD of 2.5pg/mL for PfHRP2. A microfluidic microplate-based immunoassay by Lee et al [38] demonstrated an LOD of 0.025 pg/µL (equivalent to 25 pg/mL) for pLDH. The ultrasensitive antigen test published by Mpina et al [20] in 2022 can detect a few parasites/μL. Although our TN-cyclon™ system produced results comparable to these previously reported advanced methods, our method can be readily implemented by adding only a few chemicals to conventional ELISA.

The pRBCs in iRPMI medium were measured, achieving statistical LODs of 2.57 × 10^−5^% (4.44 × 10^−2^ parasites/µL) for the PfHRP2 detection system and 2.41 × 10^−5^% (3.62 × 10^−2^ parasites/µL) for the pLDH detection system. Additionally, for pseudo-malaria-infected blood samples, both the PfHRP2 and pLDH detection systems demonstrated significant signal differences compared to non-parasitized blood. We compared these results with other reported methodologies. The droplet digital PCR method by Vera-Arias et al [39] demonstrated a theoretical LOD of 0.33 parasites/µL. The cell microarray chip system developed by Yatsuhiro et al [40] successfully detected parasites from patient samples with a parasitemia as low as 0.0001%. The flow cytometry method utilizes DNA-binding fluorescent dyes to detect the nuclei of malaria parasites and quantify infected cells, achieving a detection limit range of 0.003%–0.01% of infected erythrocytes [41, 42]. On the other hand, PCR uses primers targeting markers such as 18S rRNA or erythrocyte membrane protein 1, allowing detection at thresholds as low as approximately 0.5 parasites/µL [43, 44].

Furthermore, because our method enables measurement in the presence of whole blood, it has the potential to be effectively applied in clinical settings. Our method also produced a significant signal from samples containing as few as 0.5 parasites/µL, demonstrating detection sensitivity comparable to that of the other methods described here. These findings indicate that our method is comparable with previously reported techniques in terms of its ability to detect low-level parasitemia.

In recent years, *P falciparum*, which lacks the HRP2 and/or HRP3 gene, has emerged. These HRP2- and/or HRP3-deficient parasites were first reported in 2010 from the Amazon region of Peru, where it was revealed that these parasites escape detection by HRP2-based RDTs [45]. Subsequently, these gene-deficient parasites have been reported in Africa, including Ethiopia, Eritrea, Djibouti, Sudan, and South Africa [46]. In 2024, they were further reported in the Lake Victoria region of Kenya [47], southern Ethiopia [48], Cameroon [49], and India [50]. The genes coding for HRP2 and HRP3 are located on separate chromosomes within the *P falciparum* genome [51], but due to the conserved repetitive epitopes shared between these 2 antigens, certain antibodies cross-react with both HRP2 and HRP3 [52]. As a result, *P falciparum*, which is deficient in HRP2 only or HRP3 only, are still detectable using HRP2-based RDTs [53]. The WHO predicts that strains with HRP2 and HRP3 mutations are likely to spread and become more prevalent and recommends switching from HRP2-based RDTs to non-HRP2-based tests, such as pLDH-based RDTs, in regions where the prevalence of HRP2/HRP3 mutations exceeds 5% [34]. Therefore, it would be useful to detect both PfHRP2 and pLDH simultaneously, as in our experiments. Our future work is to examine detection using RBCs infected with HRP2-deficient parasites.

As described above, the present study has several limitations. Patient samples were not evaluated, and therefore, a reduction in detection sensitivity may occur when the assay is applied to clinical specimens. In its current form, the method is not yet directly applicable as an RDT. Although the basic principle is similar to that of a standard ELISA, efforts are underway to develop an automated, simplified, and portable system that operates on battery power and can perform measurements even under non-temperature-controlled conditions. The present assay is based on a sandwich ELISA combined with an enzyme cycling system, a concept that will be retained in the future RDT format. The method utilizes visible light at 400 nm rather than ultraviolet light, fluorescence, or luminescence, providing advantages in safety and equipment simplicity. Overall, the present study represents an initial step toward the development of a practical, field-deployable diagnostic platform.

As described in the “Introduction,” a direct comparison between nucleic acid–based and antigen-based detection limits is not straightforward, because the target analytes and their clinical implications differ. Therefore, the present study focused on antigens that reflect the presence of viable pathogens [54]. Future work will aim to translate the concept demonstrated here into practical applications. In conclusion, our newly developed detection method, TN-cyclon™, is an ELISA that does not require expensive equipment or complicated methods and utilizes a visible light color reaction. Furthermore, the combined detection of PfHRP2 and pLDH antigens may enhance diagnostic accuracy, particularly in cases involving antigen deletion or low parasite density. Continued development of a simplified, field-deployable ELISA device is expected to facilitate the use of this technology beyond research laboratories and tertiary hospitals, ultimately contributing to malaria diagnosis in low- and middle-income countries.
